# Synergistic decrease of DNA single-strand break repair rates in mouse neural cells lacking both Tdp1 and aprataxin

**DOI:** 10.1016/j.dnarep.2009.02.002

**Published:** 2009-06-04

**Authors:** Sherif F. El-Khamisy, Sachin Katyal, Poorvi Patel, Limei Ju, Peter J. McKinnon, Keith W. Caldecott

**Affiliations:** a Genome Damage and Stability Centre, University of Sussex, Brighton, BN1 9RQ, UK; b Biochemistry Department, Faculty of Pharmacy, Ain Shams University, Cairo, Egypt; c Department of Genetics, St Jude Children's Research Hospital, Memphis, TN, USA

**Keywords:** Aprataxin, Tdp1, Single-strand breaks, Neural cells, Oxidative stress, Topoisomerase-1

## Abstract

Ataxia oculomotor apraxia-1 (AOA1) is an autosomal recessive neurodegenerative disease that results from mutations of aprataxin (*APTX*). APTX associates with the DNA single- and double-strand break repair machinery and is able to remove AMP from 5′-termini at DNA strand breaks *in vitro*. However, attempts to establish a DNA strand break repair defect in APTX-defective cells have proved conflicting and unclear. We reasoned that this may reflect that DNA strand breaks with 5′-AMP represent only a minor subset of breaks induced in cells, and/or the availability of alternative mechanisms for removing AMP from 5′-termini. Here, we have attempted to increase the dependency of chromosomal single- and double-strand break repair on aprataxin activity by slowing the rate of repair of 3′-termini in aprataxin-defective neural cells, thereby increasing the likelihood that the 5′-termini at such breaks become adenylated and/or block alternative repair mechanisms. To do this, we generated a mouse model in which APTX is deleted together with tyrosyl DNA phosphodiesterase (TDP1), an enzyme that repairs 3′-termini at a subset of single-strand breaks (SSBs), including those with 3′-topoisomerase-1 (Top1) peptide. Notably, the global rate of repair of oxidative and alkylation-induced SSBs was significantly slower in *Tdp1*^−/−^/*Aptx*^−/−^ double knockout quiescent mouse astrocytes compared with *Tdp1*^−/−^ or *Aptx*^−/−^ single knockouts. In contrast, camptothecin-induced Top1-SSBs accumulated to similar levels in *Tdp1*^−/−^ and *Tdp1*^−/−^/*Aptx*^−/−^ double knockout astrocytes. Finally, we failed to identify a measurable defect in double-strand break repair in *Tdp1*^−/−^, *Aptx*^−/−^ or *Tdp1*^−/−^/*Aptx*^−/−^ astrocytes. These data provide direct evidence for a requirement for aprataxin during chromosomal single-strand break repair in primary neural cells lacking Tdp1.

## 1 Introduction

Defects in the appropriate response to DNA damage have been associated with several neurological disorders [1]. The most common form of DNA damage is that produced in one strand of the double helix, resulting in DNA single-strand breaks (SSBs). One of the most frequent sources of SSBs is oxidative attack of endogenous reactive oxygen species (ROS) on DNA [2]. In addition, SSBs can also arise during the enzymatic activities of DNA topoisomerase-1 (Top1) or DNA ligase. The abortive activity of the latter is associated with one of the most common ataxias, ataxia oculomotor apraxia-1 (AOA1). This recessive disease resembles the archetypal DNA damage-associated syndrome, ataxia telangiectasia (A-T), in neurological phenotype, but lacks the extra-neurological features of A-T such as immunodeficiency and cancer predisposition [3–5]. The gene that is mutated in AOA1, *APTX*, encodes the protein aprataxin (APTX). Based on sequence alignments, it has been shown that aprataxin belongs to the histidine triad superfamily of nucleotide hydrolases/transferases [6]. Consistent with this, aprataxin can remove adenosine monophosphate (AMP) from various AMP-linked substrates *in vitro* [7]. However, the most robust substrate so far reported for aprataxin is adenylated DNA, in which AMP-linked to the 5′-terminus of single- or double-stranded DNA breaks, suggesting that this may be the physiological substrate for this enzyme [8].

Adenylated DNA breaks are normal intermediates of DNA ligation reactions, and are usually very transient, because DNA ligases rapidly catalyse nucleophilic attack of the 5′-AMP by the 3′-hydroxyl terminus, releasing AMP and sealing the nick. However, in cases of DNA damage most 3′-termini lack a hydroxyl group and so require repair by 3′-end processing enzymes before DNA ligation can occur. Failure to do so may result in premature adenylation of the 5′-terminus and persistence of 5′-AMP, which is subsequently removed by aprataxin [9]. Aprataxin may thus reverse premature adenylation reactions at DNA breaks in a reaction that involves a covalent AMP-APTX intermediate [10].

SSBs can also arise from the abortive activity of DNA topoisomerase-1, which results in SSBs whereby Top1 is covalently attached to the 3′-terminus. Top1 peptides are removed from 3′-termini by TDP1, mutation of which is responsible for the neurodegenerative disease, spinocerebellar ataxia with axonal neuropathy-1 (SCAN1) [11]. SCAN1 patients and Tdp1, knockout mice exhibit late onset cerebellar degeneration, pointing at an important role for TDP1 in non-dividing neural cells [12]. Top1 associated DNA breaks can arise by collision of cleavage complex intermediates of Top1 activity, with RNA or DNA polymerases [13,14]. Consequently, TDP1-deficient human cells and *Tdp1*^−/−^ mouse neural cells exhibit a pronounced defect in the repair of SSBs induced by genotoxic agents that prolong the half-life of cleavage complexes, including the Top1-inhibitor camptothecin (CPT) and oxidative DNA damage [12,15–18]. The latter is partly due to Top1-SSBs induced by oxidative DNA damage [19].

In contrast to SCAN1 where there is a clear defect in cellular SSB repair (SSBR), attempts to demonstrate a defect in DNA strand break repair in AOA1 have proved conflicting and unclear. For example, it has been reported that aprataxin is recruited at sites of SSBs in an Xrcc1-dependent manner and that SSBR is impaired in aprataxin-knockdown cells [20]. A defect in cellular SSBR has also been reported in AOA1 cells in some studies [21,22], however, no evidence for such a defect was reported in others [23]. Consistent with the latter results, our experiments with APTX knockout chicken DT40 cells, *APTX*^−/−^ mouse fibroblasts, AOA1 human fibroblasts, and AOA1 lymphobastoid cells also failed to detect a measurable defect in global SSBR rates [24]. We reasoned that the lack of consistent or clear defect in SSBR in aprataxin-defective cells may reflect that breaks with 5′-AMP arise at low frequency, and/or the availability of other factors for repair of 5′-AMP termini. To investigate these possibilities further we have employed a genetic approach to examine the impact of aprataxin on chromosomal SSBR rates, using mouse models in which aprataxin was deleted either alone or in combination with deletion of the 3′-end processing enzyme Tdp1. We report that the rate of repair of oxidative and alkylation-induced single-strand breaks is reduced in *Tdp1*^−/−^/*Aptx*^−/−^ double knockout astrocytes, compared with *Tdp1*^−/−^ or *Aptx*^−/−^ single knockouts. These data provide direct evidence for an involvement of aprataxin in chromosomal SSBR in primary neural cells.

## 2 Results

**Fig. 1:**
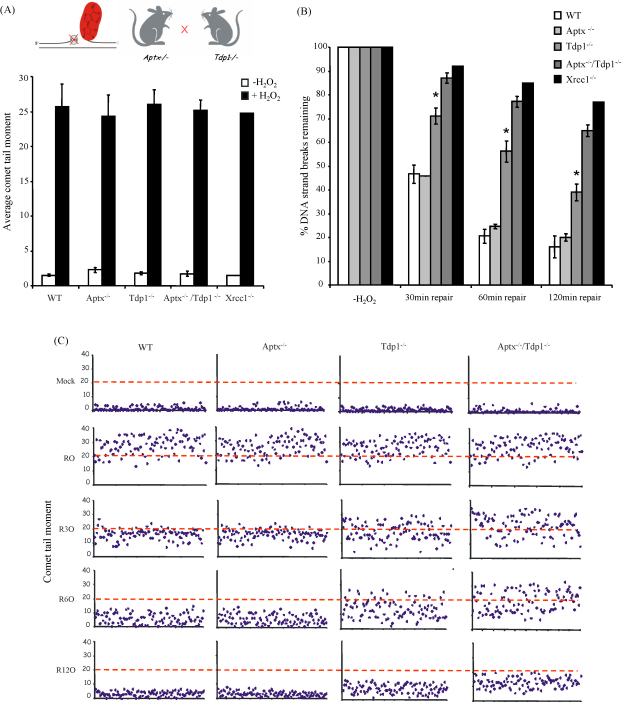
Deletion of *Tdp1* in *Aptx*^−/−^ neural cells uncovers a requirement for aprataxin during repair of “oxidative” DNA strand breaks. (A) Primary quiescent cortical astrocytes from wild-type, *Aptx*^−/−^, *Tdp1*^−/−^, and *Tdp1*^−/−^/*Aptx*^−/−^ littermates were mock treated (−H_2_O_2_) or treated (+H_2_O_2_) with 75 μM H_2_O_2_ and levels of DNA strand breakage were quantified by alkaline comet assays. *Inset*: Cartoon depicting the approach employed here to increase the sub-fraction of 5′-AMP breaks, by slowing the rate of repair of 3′-termini at SSBs via co-deletion of Tdp1 in aprataxin-defective neural cells. (B) Following treatment with H_2_O_2_ as indicated above, cells were incubated for the indicated repair periods in drug-free medium and mean fraction of DNA strand breaks remaining at the indicated time points were quantified by alkaline comet assays. Mean tail moments were quantified for 100 cells/sample/experiment and data are the average for three independent experiments (±S.E.M.). Asterisks denote statistically significant (*P* < 0.05; *t*-test) differences between *Tdp1*^−/−^ and *Tdp1*^−/−^/*Aptx*^−/−^ histograms at the indicated time points (C) Representative scatter plots from one of the experiments included in (B), showing comet tail moments of 100 individual cells per sample at the time points indicated.

To examine the functional interaction between Tdp1 and Aptx during SSBR we generated a mouse in which both genes were disrupted, by interbreeding *Tdp1*^−/−^ and *Aptx*^−/−^ mice (Fig. 1A, inset). Repair assays were then conducted on quiescent primary cortical astrocytes from wild-type, *Tdp1*^−/−^, *Aptx*^−/−^, and *Tdp1*^−/−^/*Aptx*^−/−^ littermates following treatment with H_2_O_2_, and level of chromosomal SSBs quantified by alkaline comet assays. Astrocytes lacking the SSBR scaffold protein Xrcc1 were also employed in parallel for comparison. As expected, and in agreement with previous reports, H_2_O_2_-induced similar levels of SSBs in each type of astrocytes (Fig. 1A). However, whereas the kinetics of removal of these breaks was delayed in *Tdp1*^−/−^ and *Xrcc1*^−/−^ cells, the rate of SSBR in quiescent Aptx^−/−^ astrocytes was similar to wild-type (Fig. 1B). Surprisingly, however, the combined loss of Aptx and Tdp1 delayed the kinetics of SSBR more than loss of Tdp1 alone. For example, during a 30 min repair period the level of H_2_O_2_-induced SSBs in wild-type and *Aptx*^−/−^ cells declined by ∼50% and that in *Tdp1*^−/−^ by 30%, whereas in *Tdp1*^−/−^/*Aptx*^−/−^ cells this level declined by only ∼10%. By the end of the 120 min repair period *Aptx*^−/−^ cells repaired ∼90% of their breaks, whereas *Tdp1*^−/−^/*Aptx*^−/−^ cells only repaired 30%. The SSBR defect of oxidative breaks in *Tdp1*^−/−^/*Aptx*^−/−^ double knockout cells, as compared to either *Tdp1*^−/−^ or *Aptx*^−/−^ single knockouts, was evident not only from the mean tail moments from multiple, averaged, experiments, but also from scatter plots of the raw data from individual experiments (Fig. 1C). Similar results were observed in cerebellar granule neurons lacking both Tdp1 and Aptx (data not shown). It should be noted that although the alkaline comet assay measures both SSBs and DSBs, >99% of breaks induced by H_2_O_2_ are SSBs and therefore under the conditions used the assay measures rates of SSBR. We conclude from these experiments that the global rate of repair of H_2_O_2_-induced SSBs is decreased in *Tdp1*^−/−^/*Aptx*^−/−^ neural cells.

**Fig. 2:**
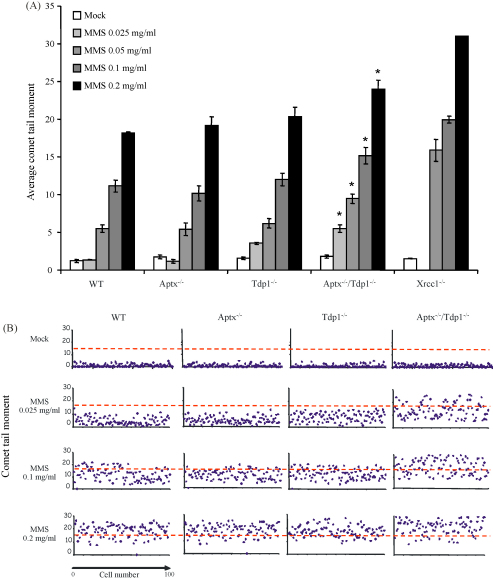
Defective repair of alkylation-induced DNA single-strand breaks in *Tdp1*^−/−^/*Aptx*^−/−^ double knockout neural cells. (A) Primary quiescent cortical astrocytes of the indicated genotypes were mock treated or treated with the indicated concentrations of MMS for 10 min at 37 °C. DNA strand breakage was then quantified by alkaline comet assays and data for 100 cells/sample/experiment were averaged from three independent experiments (±S.E.M.). Asterisks denote statistically significant (*P* < 0.05; *t*-test) differences between wild-type and *Tdp1*^−/−^/*Aptx*^−/−^ histograms. (B) Representative scatter plots from one of the experiments included in (A).

Next, we examined the repair of SSBs in *Tdp1*^−/−^/*Aptx*^−/−^ double knockout astrocytes induced by the alkylating agent MMS. During the course of repair of base damage a 5′-deoxyribose phosphate (dRp) intermediate is formed, which can be subsequently adenylated by DNA ligase [25]. We measured the level of DNA strand breakage present following 10 min incubations with different concentrations of MMS, which reflects the steady state level of SSBs and abasic sites during base excision repair. Similar levels of SSBs were present in wild-type, *Tdp1*^−/−^, and *Aptx*^−/−^ cells following 10 min incubations with different concentrations of MMS, though a significantly more SSBs were present in *Tdp1*^−/−^ cells at the lowest concentration (Fig. 2A). However, the level of SSBs that accumulated in *Tdp1*^−/−^/*Aptx*^−/−^ double knockout astrocytes was significantly higher than in either WT, *Tdp1*^−/−^, *or Aptx*^−/−^ astrocytes at all MMS concentrations examined (Fig. 2A). The elevated steady state level of SSBs present in *Tdp1*^−/−^/*Aptx*^−/−^ double knockout astrocytes was evident from the average tail moment of multiple experiments and from the raw data of individual experiments (Fig. 2B). We conclude from these experiments that *Tdp1*^−/−^/*Aptx*^−/−^ double knockout astrocytes exhibit reduced rates of SSB rejoining during the base excision repair of alkylation DNA damage.

**Fig. 3:**
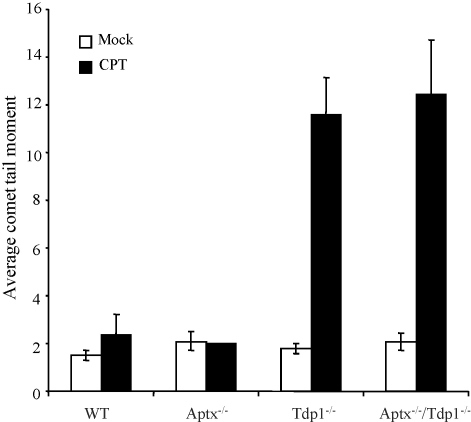
Accumulation of similar levels of camptothecin-induced DNA single-strand breaks in *Tdp1*^−/−^ and *Tdp1*^−/−^/*Aptx*^−/−^ double knockout neural cells. Primary quiescent cortical astrocytes were mock treated or treated with 14 μM camptothecin (CPT) for 1 h at 37 °C and DNA strand breakage was then quantified by alkaline comet assays. Mean tail moments were plotted ±S.E.M.

We next compared the induction and repair of SSBs in quiescent astrocytes following treatment with camptothecin, a SSB-inducing agent that generates breaks with Top1-associated 3′-termini and 5′-hydroxyl termini. As reported previously, high levels of SSBs accumulated in *Tdp1*^−/−^ cells during a 1-h incubation with CPT, due to a defect in chromosomal SSBR (Fig. 3). In contrast, SSBs failed to accumulate in *Aptx*^−/−^ astrocytes above that observed in wild-type cells, and the level of SSBs that accumulated in *Tdp1*^−/−^/*Aptx*^−/−^ double knockout astrocytes was not more than that in *Tdp1*^−/−^ single knockouts (Fig. 3). We conclude from these experiments that the repair of CPT-induced DNA SSBs in *Tdp1*^−/−^ astrocytes is not affected by the additional deletion of *Aptx*.

**Fig. 4:**
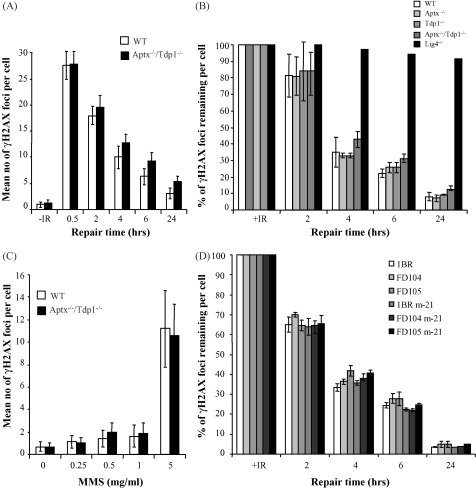
Normal rates of DNA double-strand break repair in *Tdp1*^−/−^/*Aptx*^−/−^ neural cells. Primary quiescent cortical astrocytes from wild-type and *Tdp1*^−/−^/*Aptx*^−/−^ littermates (A) or quiescent wild-type (1BR), h-Tert immortalized AOA1 (FD104 and FD105), and AOA1 fibroblasts stably expressing recombinant human aprataxin (FD104 m-21 and FD105 m-21) (D) were mock (0) or γ-irradiated (3Gy) on ice and then incubated for the indicated repair periods at 37 °C (A, B, and D) or incubated with the indicated concentrations of MMS for 10 min at 37 °C (C). The mean number of γH2AX foci was quantified from 50 cells/sample/experiment and data represent the average of three independent experiments (±S.E.M.). (B) The mean fraction of γ-ray-induced γH2AX foci remaining at the indicated repair periods were quantified ±S.E.M. *Lig4*^−/−^ mouse embryonic fibroblasts were included in parallel for comparison.

Since 5′-AMP can most likely arise at both single- and double-strand breaks, we next examined whether *Aptx*^−/−^ and *Tdp1*^−/−^/*Aptx*^−/−^ neural cells possess a defect in DSBR. For these experiments we employed ionizing radiation (IR), which also induces oxidative breaks but with a greater ratio of DSBs to SSBs (∼1:25) than does H_2_O_2_ (∼1:2000). We first compared quiescent wild-type and *Tdp1*^−/−^/*Aptx*^−/−^ astrocytes for formation of γ-H2AX foci, an established marker for DSBs, following treatment with IR. The number and intensity of γ-H2AX foci formed after IR were similar in wild-type and *Tdp1*^−/−^/*Aptx*^−/−^ astrocytes, and there was no significant difference (*P* > 0.1 at all time points examined) in the rate at which these foci disappeared during subsequent repair incubations (Fig. 4A). To confirm these observations, we compared *Tdp1*^−/−^/*Aptx*^−/−^ double-knockout with *Tdp1*^−/−^, *Aptx*^−/−^ single knockout astrocytes, and *Lig4*^−/−^ mouse embryonic fibroblasts, for the level of IR-induced γ-H2AX foci. Whereas the initial number of induced DSBs and the kinetics of their removal during a subsequent 24 h repair period were similar in wild-type, *Tdp1*^−/−^, *Aptx*^−/−^, and *Tdp1*^−/−^/*Aptx*^−/−^ astrocytes, we observed that *Lig4*^−/−^ cells exhibited severely delayed γH2AX foci removal kinetics, consistent with their established cellular defect in DSBR (Fig. 4B). Similar results were observed following treatment of quiescent astrocytes with MMS, which in the absence of ongoing DNA replication can produce DSBs at high drug concentrations by generating overlapping base damage on opposite DNA strands (Fig. 4C). Finally, we also failed to detect a difference between the rate of disappearance of IR-induced γ-H2AX foci in quiescent h-Tert immortalized AOA1 fibroblasts and either wild-type controls or AOA1 fibroblasts stably expressing recombinant human aprataxin (Fig. 4D). We conclude from these experiments that global rates of DSBR are normal in aprataxin-deficient cells, following IR.

In summary, co-deletion of Tdp1 and Aptx uncovers a requirement for aprataxin for rapid rates of chromosomal SSBR, but not DSBR, in quiescent primary neural cells following oxidative or alkylation DNA damage.

## 3 Discussion

AOA1 is a recessive hereditary spinocereballar ataxia resulting from mutations in aprataxin, a component of DNA strand break repair machinery [3,4]. Cell free assays showed that APTX removes AMP from 5′-termini at DNA strand breaks [8,10,25]. However, whether 5′-AMP DNA breaks are the physiological substrates for aprataxin has not been determined. In addition, attempts to detect a defect in DNA single- and/or double-strand break repair in aprataxin-defective cells have proved conflicting and unclear [8,20–22]. Here, we report that global rates of chromosomal SSBR are normal in quiescent *Aptx*^−/−^ neural cells, including astrocytes and cerebellar granule neurons. Similar results were observed with a variety of other aprataxin-defective cells, including Aptx^−^ chicken DT40 cells, *Aptx*^−/−^ mouse embryonic fibroblasts, and AOA1 primary human fibroblasts [24]. However, we describe here a synergistic decrease in SSBR rates in *Aptx*^−/−^ cells in which Tdp1, a 3′-end processing enzyme that is mutated in the neurodegenerative disease SCAN1, is also deleted. Indeed, in the case of oxidative SSBs, the SSBR defect in *Aptx*^−/−^/*Tdp1*^−/−^ double knockout astrocytes approached that of astrocytes lacking the core SSBR scaffolding factor, Xrcc1.

There are several possible explanations for the synergistic impact of Tdp1 and Aptx deletion on SSBR. First, the fraction of cellular breaks harbouring 5′-AMP may be too small to detect in Aptx^−/−^ single mutants, using global repair assays. This is because the likely source of 5′-AMP SSBs is premature DNA adenylation by DNA ligases, the occurrence of which will depend on the half-life of the SSB and thus the rate of processing of the 3′-terminus. Consequently, the additional loss of 3′-end processing factors such as Tdp1 may increase the cellular level of 5′-AMP aprataxin substrates. In this scenario, the absence of a synergistic impact of Aptx and Tdp1 co-deletion on SSBR rates following CPT in non-cycling cells might be explained by the absence of a 5′-phosphate terminus at CPT-induced SSBs, which is required for 5′-DNA adenylation. Alternatively, in Aptx^−/−^ cells, cellular SSBs harbouring 5′-AMP may be repaired by one or more aprataxin-independent pathways. For example, the 5′-AMP DNA may be excised by a structure specific nuclease such as XPG or MRN followed by gap filling and ligation [26]. An alternative mechanism may involve long-patch SSBR, in which 5′-AMP termini are displaced as part of a single-stranded flap by extended gap-filling and removed by Flap endonuclease-1 (FEN1). In this scenario, the slower repair in Aptx^−/−^/Tdp1^−/−^ double mutants, compared to Tdp1^−/−^ mutants, may reflect a delay in long-patch repair imparted by loss of Tdp1, which may slow restoration of a 3′-hydroxyl primer-terminus at the break. In support of this notion, we have recently uncovered a single-strand breaks repair defect in Aptx^−/−^ quiescent astrocytes by inhibiting long-patch DNA polymerases [24]. In this scenario, it is plausible to speculate that the presence of 5′-AMP may impede the repair of the 3′-terminus by other Tdp1-independent processes. Indeed, we have evidence for the existence of such mechanisms in non cycling cells (El-Khamisy, unpublished observations).

In contrast to SSBR, we failed to detect a defect in DSBR in either Tdp1 or Aptx single mutants or in *Tdp1*^−/−^/*Aptx*^−/−^ double mutants, as measured using γH2AX assays. However, aprataxin is associated with the DSBR machinery and it is likely that 5′-AMP can arise at DSBs. It is possible that the fraction of DSBs possessing 5′-AMP falls below the limit of detection, even in the absence of both Aptx and Tdp1. Alternatively, there may exist in cells an aprataxin-independent mechanisms for removing 5′-AMP at DSBs that are unaffected by additional deletion of Tdp1. The availability of structure specific nucleases for trimming damaged 5′-termini at DSBs is one such possibility.

Whether or not SSBs and/or DSBs contribute to the neurological pathology of AOA1 remains to be determined. We have demonstrated here that aprataxin is required for normal rates of SSBR under specific experimental conditions, but that loss of aprataxin alone does not markedly impact on global rates of SSBR or DSBR. It is possible that specific types of human neurons form higher levels of 5′-AMP SSBs than those measured here in *Aptx*^−/−^ quiescent astrocytes or that alternative mechanisms for removing 5′-AMP independently of aprataxin are unavailable in such cells *in vivo*. For example, it has been suggested that long-patch repair is attenuated in some types of post-mitotic cells [27], raising the possibility that the cells affected in AOA1 are more reliant on aprataxin-dependent short-patch pathway. It should be possible to address this and other hypotheses by measuring SSBR and DSBR capacity in the specific neuronal cell types that are affected in AOA1.

In summary, the data presented here provide evidence that APTX is operative at chromosomal SSBs in primary neural cells. It will now be important to understand whether specific neuronal cell populations exhibit greater dependence on aprataxin for chromosomal SSBR and/or DSBR, and to determine whether DNA breaks with 5′-AMP are the physiological substrate for this enzyme, *in vivo*.

## 4 Materials and methods

### 4.1 Generation of the *Tdp1*^−/−^/*Aptx*^−/−^ double knockout mice

The generation of *Tdp1*^−/−^ and *Aptx*^−/−^ single knockout mice was described previously [8,12]. *Tdp1*^+/−^ and *Aptx*^+/−^ mice were bred to generate *Tdp1*^+/−^, *Aptx*^+/−^ double heterozygote animals, which were then interbred to generate *Tdp1*^−/−^, *Aptx*^−/−^ double knockout mice. *Tdp1*^+/−^, *Aptx*^+/−^ double heterozygote animals were maintained on an outbred mixed 129Ola and C57BL/6 background. Genotyping for the mutant *Tdp1* and *Aptx* alleles was performed as described [8,12]. All animals were housed in individually ventilated cabinets in the school of life science and were maintained in accordance with the institutional animal care and ethical committee at the University of Sussex.

### 4.2 Cell culture

#### 4.2.1 Human cells

AOA1 primary fibroblasts were immortalized by h-Tert, following standard protocols. The hTert immortalized AOA1 fibroblasts (FD104 and FD105) and wild-type controls (1BR) were then stably transfected with plasmids encoding for recombinant human aprataxin, generating FD104 m-21, FD105 m21, and 1BR m-21. Fibroblasts were cultured in minimal essential medium supplemented with 15% foetal calf serum (FCS), l-glutamine, penicillin and streptomycin.

#### 4.2.2 Primary mouse astrocytes

Mouse astrocytes were prepared from P3 to P4 brains. Cortices were dissociated by passage through a 5 ml pipette, cells were resuspended in Dulbecco's modified Eagle's medium and Ham's nutrient mixture F-12 (1:1 DMEM/F12; Gibco-BRL) supplemented with 10% fetal bovine serum, 1× glutamax, 100 U/ml penicillin, 100 μg/ml streptomycin, and 20 ng/ml epidermal growth factor (Sigma). Primary astrocytes were established in Primeria T-25 tissue culture flasks (Falcon) at 37 °C in a humidified oxygen-regulated (5%) incubator. Culture medium was changed after 3 days and astrocyte monolayers reached confluence 3 days later. The purity of the culture was confirmed by immunofluorescence using an anti-GFAP antibody (Sigma), a marker for astrocytes.

### 4.3 Single-strand breaks repair assays

Quiescent astrocytes (∼3 × 10^5^ cells/sample) were incubated with 75 μM H_2_O_2_ for 10 min on ice, with the indicated doses of methyl methanesulfonate (MMS) for 10 min at 37 °C, or with 14 μM camptothecin for 60 min at 37 °C. Where indicated, cells were then incubated in drug-free medium for the indicated repair periods at 37 °C. Chromosomal DNA strand breaks were then measured using alkaline comet assays as previously described [3,4], with few modifications. Briefly, cells were suspended in pre-chilled PBS and mixed with equal volume of 1.2% low-gelling-temperature agarose (Sigma, type VII) maintained at 42 °C. The cell suspension was immediately layered onto pre-chilled frosted glass slides (Fisher) pre-coated with 0.6% agarose and maintained in the dark at 4 °C until set, and for all further steps. Slides were immersed in pre-chilled lysis buffer (2.5 M NaCl, 10 mM Tris–HCl, 100 mM EDTA, 1% Triton X-100, 10% DMSO, pH 10) for 1 h, washed with pre-chilled distilled water (2× 10 min), and placed for 45 min in pre-chilled alkaline electrophoresis buffer (50 mM NaOH, 1 mM EDTA, 10% DMSO). Electrophoresis was then conducted at 25 V (0.6 V/cm) for 25 min, followed by neutralization in 400 mM Tris–HCl pH 7.0 for 1 h. Finally, DNA was stained with Sybr Green I (1:10,000 dilution in PBS) for 30 min. DNA strand breakage was expressed as “comet tail moment”, which is the product of the tail length and the fraction of DNA that has exited the nucleus during electrophoresis [28]. The comet tail moment was measured for at least 50 cells per sample using Comet Assay IV software (Perceptive Instruments, UK).

### 4.4 Double-strand breaks repair assays

Quiescent fibroblasts or astrocytes were grown on coverslips and then exposed to γ-irradiation (3Gy) using a cesium-137 source (CammaeII 1000) in complete medium on ice, or to the indicated concentrations of MMS (∼10-fold the doses used for measuring SSBs) for 10 min at 37 °C. Where indicated, cells were then incubated in complete medium for the indicated repair periods at 37 °C. Cells were then fixed with 4% PFA/PBS for 10 min at room temperature, followed by incubation with 0.5% Triton X-100/PBS for 5 min on ice. Cells were rinsed with PBS and incubated with 5% BSA for 30 min at 37 °C to block non-specific binding, followed by incubation with mouse anti-γH2AX monoclonal antibodies (Upstate; clone JBW301, 1/800 dilution in 3%BSA) for 30 min at 37 °C. After rinsing with PBS, cells were incubated in FITC-conjugated anti-mouse IgG (DAKO) secondary antibodies at 1:200 dilution in 4% BSA for 30 min at 37 °C. Nuclei were counterstained with 0.000025% DAPI. γH2AX foci were counted for at least 50 cells per time point.

### 4.5 Statistical analysis

Statistical analyses were performed using SPSS software. Differences were considered significant when *P* value was less than 0.05.

## Conflict of interest

The authors declare that there are no conflicts of interest.

## References

[1] Katyal S., McKinnon P.J. (2008). DNA strand breaks, neurodegeneration and aging in the brain. Mech. Ageing Dev..

[2] Caldecott K.W. (2008). Single-strand break repair and genetic disease. Nat. Rev. Genet..

[3] Date H., Onodera O., Tanaka H., Iwabuchi K., Uekawa K., Igarashi S., Koike R., Hiroi T., Yuasa T., Awaya Y., Sakai T., Takahashi T., Nagatomo H., Sekijima Y., Kawachi I., Takiyama Y., Nishizawa M., Fukuhara N., Saito K., Sugano S., Tsuji S. (2001). Early-onset ataxia with ocular motor apraxia and hypoalbuminemia is caused by mutations in a new HIT superfamily gene. Nat. Genet..

[4] Moreira M.C., Barbot C., Tachi N., Kozuka N., Uchida E., Gibson T., Mendonca P., Costa M., Barros J., Yanagisawa T., Watanabe M., Ikeda Y., Aoki M., Nagata T., Coutinho P., Sequeiros J., Koenig M. (2001). The gene mutated in ataxia-ocular apraxia 1 encodes the new HIT/Zn-finger protein aprataxin. Nat. Genet..

[5] Lavin M.F., Gueven N., Grattan-Smith P. (2008). Defective responses to DNA single- and double-strand breaks in spinocerebellar ataxia. DNA Repair (Amst.).

[6] Kijas A.W., Harris J.L., Harris J.M., Lavin M.F. (2006). Aprataxin forms a discrete branch in the HIT (histidine triad) superfamily of proteins with both DNA/RNA binding and nucleotide hydrolase activities. J. Biol. Chem..

[7] Seidle H.F., Bieganowski P., Brenner C. (2005). Disease-associated mutations inactivate AMP-lysine hydrolase activity of Aprataxin. J. Biol. Chem..

[8] Ahel I., Rass U., El-Khamisy S.F., Katyal S., Clements P.M., McKinnon P.J., Caldecott K.W., West S.C. (2006). The neurodegenerative disease protein aprataxin resolves abortive DNA ligation intermediates. Nature.

[9] Rass U., Ahel I., West S.C. (2007). Defective DNA repair and neurodegenerative disease. Cell.

[10] Rass U., Ahel I., West S.C. (2008). Molecular mechanism of DNA deadenylation by the neurological disease protein aprataxin. J. Biol. Chem..

[11] Takashima H., Boerkoel C.F., John J., Saifi G.M., Salih M.A., Armstrong D., Mao Y., Quiocho F.A., Roa B.B., Nakagawa M., Stockton D.W., Lupski J.R. (2002). Mutation of TDP1, encoding a topoisomerase I-dependent DNA damage repair enzyme, in spinocerebellar ataxia with axonal neuropathy. Nat. Genet..

[12] Katyal S., El-Khamisy S.F., Russell H.R., Li Y., Ju L., Caldecott K.W., McKinnon P.J. (2007). TDP1 facilitates chromosomal single-strand break repair in neurons and is neuroprotective in vivo. EMBO J..

[13] Strumberg D., Pilon A.A., Smith M., Hickey R., Malkas L., Pommier Y. (2000). Conversion of topoisomerase I cleavage complexes on the leading strand of ribosomal DNA into 5′-phosphorylated DNA double-strand breaks by replication runoff. Mol. Cell. Biol..

[14] Wu J., Liu L.F. (1997). Processing of topoisomerase I cleavable complexes into DNA damage by transcription. Nucleic Acids Res..

[15] Hirano R., Interthal H., Huang C., Nakamura T., Deguchi K., Choi K., Bhattacharjee M.B., Arimura K., Umehara F., Izumo S., Northrop J.L., Salih M.A., Inoue K., Armstrong D.L., Champoux J.J., Takashima H., Boerkoel C.F. (2007). Spinocerebellar ataxia with axonal neuropathy: consequence of a Tdp1 recessive neomorphic mutation?. EMBO J..

[16] Miao Z.H., Agama K., Sordet O., Povirk L., Kohn K.W., Pommier Y. (2006). Hereditary ataxia SCAN1 cells are defective for the repair of transcription-dependent topoisomerase I cleavage complexes. DNA Repair (Amst.).

[17] El-Khamisy S.F., Saifi G.M., Weinfeld M., Johansson F., Helleday T., Lupski J.R., Caldecott K.W. (2005). Defective DNA single-strand break repair in spinocerebellar ataxia with axonal neuropathy-1. Nature.

[18] Interthal H., Chen H.J., Kehl-Fie T.E., Zotzmann J., Leppard J.B., Champoux J.J. (2005). SCAN1 mutant Tdp1 accumulates the enzyme–DNA intermediate and causes camptothecin hypersensitivity. EMBO J..

[19] El-Khamisy S.F., Hartsuiker E., Caldecott K.W. (2007). TDP1 facilitates repair of ionizing radiation-induced DNA single-strand breaks. DNA Repair (Amst.).

[20] Hirano M., Yamamoto A., Mori T., Lan L., Iwamoto T.A., Aoki M., Shimada K., Furiya Y., Kariya S., Asai H., Yasui A., Nishiwaki T., Imoto K., Kobayashi N., Kiriyama T., Nagata T., Konishi N., Itoyama Y., Ueno S. (2007). DNA single-strand break repair is impaired in aprataxin-related ataxia. Ann. Neurol..

[21] Mosesso P., Piane M., Palitti F., Pepe G., Penna S., Chessa L. (2005). The novel human gene aprataxin is directly involved in DNA single-strand-break repair. Cell. Mol. Life Sci..

[22] Gueven N., Chen P., Nakamura J., Becherel O.J., Kijas A.W., Grattan-Smith P., Lavin M.F. (2007). A subgroup of spinocerebellar ataxias defective in DNA damage responses. Neuroscience.

[23] Gueven N., Becherel O.J., Kijas A.W., Chen P., Howe O., Rudolph J.H., Gatti R., Date H., Onodera O., Taucher-Scholz G., Lavin M.F. (2004). Aprataxin, a novel protein that protects against genotoxic stress. Hum. Mol. Genet..

[24] Reynolds J.J., El-Khamisy S.F., Katyal S., Clements P., McKinnon P.J., Caldecott K.W. (2008). Defective DNA ligation during short-patch single-strand break repair in ataxia oculomotor apraxia-1. Mol. Cell. Biol..

[25] Rass U., Ahel I., West S.C. (2007). Actions of aprataxin in multiple DNA repair pathways. J. Biol. Chem..

[26] O’Donovan A., Davies A.A., Moggs J.G., West S.C., Wood R.D. (1994). XPG endonuclease makes the 3′ incision in human DNA nucleotide excision repair. Nature.

[27] Narciso L., Fortini P., Pajalunga D., Franchitto A., Liu P., Degan P., Frechet M., Demple B., Crescenzi M., Dogliotti E. (2007). Terminally differentiated muscle cells are defective in base excision DNA repair and hypersensitive to oxygen injury. Proc. Natl. Acad. Sci. U.S.A..

[28] Olive P.L., Banath J.P., Durand R.E. (1990). Heterogeneity in radiation-induced DNA damage and repair in tumor and normal cells measured using the “comet” assay. Radiat. Res..

